# Transcriptome Sequencing Analysis Reveals the Mechanisms of Poly-γ-Glutamic Acid Enhanced the Chilling and Freezing Tolerance in Wheat

**DOI:** 10.3390/biology15030293

**Published:** 2026-02-06

**Authors:** Yuqi Niu, Jiang Liu, Bin Bu, Zhaohui Tang, Yongkang Ren, Haizhen Ma

**Affiliations:** 1 College of Agriculture, Shanxi Agricultural University, Taiyuan 030031, China; 2 School of Bioengineering, Qilu University of Technology (Shandong Academy of Sciences), Jinan 250300, China

**Keywords:** γ-PGA, wheat, chilling and freezing stress, RNA-seq, cold resistance

## Abstract

Low-temperature stress significantly limits wheat growth and productivity. Poly-γ-glutamic acid (γ-PGA) is an environmentally friendly green molecular material that plays an important role in plant growth and regulation; however, its protective mechanisms against cold stress in wheat remain poorly understood. In this study, the effect of γ-PGA on both chilling (4 °C) and freezing (−18 °C) resistance in wheat seedlings and its underlying mechanisms were comparatively studied. The results showed that the γ-PGA-treated seedlings exhibited a 128.81% higher survival rate after freezing stress and maintained significantly greater biomass accumulation under both stress conditions (62.44% and 26.56% higher dry weight under chilling and freezing stress, respectively). A physiological analysis revealed that γ-PGA enhanced osmoprotectant (proline and soluble sugars) accumulation and activated key antioxidant enzymes (SOD, POD, and APX). Then, an RNA-seq analysis identified 11,401 and 7721 differentially expressed genes under chilling and freezing stress, respectively, with 3598 common genes constituting a core cold-response network. KEGG and GO analyses demonstrated significant enrichment in pathways related to carbon metabolism, glutathione metabolism, phenylpropanoid–flavonoid biosynthesis, fatty acid metabolism, and cell wall organization. Notably, γ-PGA strongly upregulated key genes in phenylpropanoid–flavonoid metabolism (*TraesCS2B02G615000* and *TraesCS2B02G624400*), glutathione metabolism (*TraesCS1B02G127900*), and lipid metabolism (*TraesCS1B02G018700*). These results provide comprehensive molecular insights into γ-PGA-mediated cold tolerance and support its potential application in sustainable wheat production under low-temperature stress conditions.

## Simple Summary

γ-PGA significantly enhances the cold resistance of wheat seedlings under both chilling (4 °C) and freezing (−18 °C) stress. Physiologically, γ-PGA promoted the accumulation of osmoregulatory substances (proline and soluble sugars) and activated the antioxidant enzyme system. Transcriptome analysis revealed that γ-PGA regulates a core cold-responsive gene network, particularly upregulating key genes involved in phenylpropanoid-flavonoid metabolism, glutathione metabolism, and lipid metabolism.

## 1. Introduction

Wheat (*Triticum aestivum* L.) is one of the three major food crops globally, serving as a source of calories for one-fifth of the global population [1,2]; thus, its stable and high yield plays a crucial role in global food security. In recent decades, with continuous greenhouse gas emissions, extreme weather events have become increasingly likely, and low-temperature freezing occurs frequently, posing a significant threat to wheat production. Frost damage causes millions of tons of food losses annually, resulting in direct economic losses exceeding tens of billions of USD. As the global climate continues to change, wheat growth and development will be subject to more cold stress, making it highly important to improve wheat varieties’ cold tolerance through breeding [3].

Wheat is a crop that must undergo vernalization at low temperatures to complete its growth cycle, making it well-suited for winter [4]. However, if wheat is in the seedling stage before winter, sudden temperature drops can lead to leaf desiccation, root damage, and even seedling death, thereby affecting yield. During winter, low temperatures can damage plant tissues and inhibit growth and development in wheat with weak cold resistance [5]. During the period from nodulation to tassel emergence, wheat undergoes both vegetative and reproductive growth. Frost or “late spring frost” can cause hollow or malformed rachises in this stage, having catastrophic consequences [6]. Data show that nearly 85% of the global wheat area is affected by late spring frost every year [7], highlighting the practical importance of research on wheat cold tolerance for production.

Cold stress is mainly divided into two categories: chilling stress (0–15 °C) and freezing stress (<0 °C) [8,9]. When wheat cellular tissues are subjected to cold damage, they can be injured or even killed, primarily due to the destruction of the cell membrane structure. The stability of the cell membrane is determined by its lipid fatty acid composition, and, generally, the higher the content of unsaturated fatty acids in membrane lipids, the stronger the cold tolerance of the plant [10]. Additionally, the reactive oxygen species (ROS) accumulation and scavenging systems in plants are crucial for maintaining normal cellular functions, and normally, they are in dynamic balance. However, sustained low temperatures can disrupt this balance, accelerating ROS accumulation and generating a large number of free radicals, which can then cause physiological disturbances within the cell [11]. Peroxidase (POD), superoxide dismutase (SOD), catalase (CAT), and ascorbate peroxidase (APX) are important protective enzymes in wheat, which can scavenge excessive ROS and free radicals produced during cold stress, maintaining normal wheat growth. Low-temperature stress can disrupt the integrity of cell membranes, interfere with the normal transport of water and nutrients, and potentially lead to ice crystal formation and consequent physiological desiccation within cells, resulting in metabolic disorders. To counteract this osmotic imbalance, plants accumulate various compatible solutes (e.g., proline, soluble sugars) as osmolytes. These compounds help stabilize cellular osmotic pressure, protect protein structures and membrane systems, thereby allowing the maintenance of relatively normal basic metabolic functions. The soluble sugar content in wheat is positively correlated with cold tolerance, with sucrose and fructose playing significant roles in wheat cold tolerance [11]. The proline content is also positively correlated with wheat cold tolerance. In fact, many studies have shown that, when temperatures are low, the proline content in wheat increases, causing more water molecules to accumulate on proteins, preventing protein dehydration and deformation at low temperatures, and thus protecting wheat cells from damage [12].

Over time, plants have developed highly sophisticated systems to cope with cold stress. For instance, they activate a series of biochemical and physiological changes as well as signaling pathways within cells, such as altering the transcription of cold-responsive genes, regulating hormone levels and responses, generating reactive oxygen species (ROS) to stimulate the accumulation of compatible osmolytes and antioxidants, and reshaping epigenetic modifications across the genome [13,14]. Among these, the ICE-CBF-COR signaling pathway represents a ubiquitous and pivotal regulatory mechanism associated with cold stress tolerance in many plant species, including crops [15]. ICE1 (Inducer of CBF Expression) is a MYC-type bHLH transcription factor [16,17]. It acts as a positive regulator of CBF (C-repeat Binding Factor) gene expression, functioning upstream of CBF to directly activate its transcription. This activation subsequently initiates the expression of a series of downstream cold-responsive (COR) genes [18].

Currently, various strategies have been effectively employed to enhance wheat resistance to low-temperature stress. These include leveraging modern biotechnology (such as transgenic technology and genome editing) to directly create new varieties of high-yield, high-quality, and cold-resistant wheat, as well as applying exogenous hormones or regulatory substances such as salicylic acid, abscisic acid, trehalose, melatonin, and potassium dihydrogen phosphate to improve wheat’s cold stress resistance [19,20].

Poly-γ-glutamic acid (γ-PGA), a green polymer, is used in agriculture as a fertilizer synergist and water-retaining agent. As a plant growth regulator, it has been proven to enhance plants’ resistance to various environmental stresses. Studies have shown that exogenous application of γ-PGA can improve plants’ resistance to multiple environmental stresses, including salt [21], drought [22], and cold [23]. These effects may be achieved by activating various signaling pathways and regulating related metabolic processes. Regarding γ-PGA’s role in enhancing plant salt tolerance, it has been reported to improve wheat’s salt tolerance by alleviating ion imbalance and enhancing antioxidant capacity [21]. In terms of enhancing cold tolerance, γ-PGA enhances the cold tolerance of *Brassica napus* seedlings by regulating the Ca^2+^/CPK signaling pathway [23]. Additionally, studies have found that γ-PGA may simultaneously activate multiple signaling pathways such as H_2_O_2_, calcium signaling, brassinolide, and jasmonic acid to jointly mediate plant salt and cold tolerance [24,25]. However, most of these research results are from studies on vegetables, with limited research on wheat. Wheat may suffer from chilling (0–15 °C) and freezing stress (<0 °C) during its growth. Therefore, we investigated whether exogenous application of γ-PGA can enhance wheat’s resistance to chilling and freezing stress by analyzing its physiological and biochemical indicators and genes involved in some key cold tolerance signaling pathways. This preliminary study reveals the mechanisms by which γ-PGA enhances wheat’s chilling and freezing resistance, laying the foundation for the subsequent widespread application of γ-PGA in agriculture.

## 2. Materials and Methods

### 2.1. Frost Resistance Experiment on Wheat Sprouts

T113 wheat seeds were surface-sterilized and then inoculated onto filter paper for germination. When the wheat sprouts reached 1–2 cm in length, the seeds of each variety were further divided into two portions, with three boxes per portion. One portion received a 50 mg/L γ-PGA solution prepared with sterile water, while the other portion received an equal volume of sterile water to ensure consistent humidity in the tissue paper within both sets of boxes. The seedlings were then transferred to a 4 °C refrigerator for 12 h of cultivation, followed by 12 h of freezing at −18 °C. After removal, they were placed back in the 4 °C refrigerator for one day of thawing and finally allowed to resume growth at room temperature for three days. The total number of sprouts and the number of surviving sprouts were recorded to calculate the sprout frost recovery rate.

### 2.2. Cold and Frost Resistance Experiments at the Seedling Stage

T113 wheat seeds were selected and planted in pots, and when the seedlings reached the three-leaf stage, treatment began. T113 seedlings were divided into two groups based on variety. One group was watered with a 50 mg/L γ-PGA solution as the experimental group, while the other group was watered with an equal amount of water as the control group. All materials were placed in a 4 °C incubator (16 h light, 8 h dark) for a 30-day vernalization period, and then, growth was allowed to resume at room temperature. Subsequently, the following treatments were applied: Experiment 1: growth under normal conditions; Experiment 2: exposure to 4 °C for 24 h followed by normal growth at room temperature; Experiment 3: pretreatment at 4 °C for 24 h (with 16 h of light and 8 h of darkness), followed by freezing at −18 °C for 3 h. After freezing, the seedlings were placed in a 4 °C incubator for 24 h of thawing and then allowed to continue growing at room temperature (Experiments 2 and 3 were designed to simulate late spring coldness after wheat vernalization, as this stage represents a critical period during which wheat seedlings are particularly sensitive to low-temperature stress [26]). Leaves from wheat seedlings exposed to 4 °C for 24 h, −18 °C for 3 h, and normal growth conditions were flash-frozen in liquid nitrogen and subsequently used for physiological index measurement and RNA-seq analysis.

### 2.3. Determination of Proline and Soluble Sugars Content, and APX, POD and SOD Activity

Total soluble sugars of fresh leaves (approximately 100 mg) were extracted in boiling water for 30 min and determined using anthrone reagent with glucose as the standard, in accordance with the methods described by Yemm and Willis [27]. The proline content of the leaves was determined using the JM-09918P1 kit (Jiangsu Jingmei Biological Technology Co., Ltd (Yancheng, China)); the total peroxidase (POD) activity was determined spectrophotometrically by measuring the oxidation of guaiacol at 470 nm using the ADS-W-KY003 kit; and the ascorbic peroxidase (APX) and SOD activity was detected using ADS-W-VC005 kits (all biochemical assay kits procured from Jiangsu Aidisheng Biological Technology Co., Ltd (Yancheng, China). Three biological replicates were performed.

### 2.4. RNA Sequencing and Analysis

Total RNA sequencing and primary bioinformatic analysis were conducted by BGI Tech Solutions Co., Ltd. (Shenzhen, China) using the T7 platform (BGI-Shenzhen, China). Raw reads were quality-controlled and filtered using SOAPnuke (v1.6.5) to obtain clean reads. The clean reads were then aligned to the wheat reference genome (IWGSC RefSeq v1.0, 2018) using HISAT2 (v2.2.1), and gene expression levels were quantified with RSEM (v1.3.1). Differentially expressed genes (DEGs) were identified using DEseq2 (1.51.6) with thresholds of |Fold Change| ≥ 1 and Adjusted *p*-value ≤0.05.

Functional annotation and enrichment analysis of the DEGs were performed. Gene Ontology (GO) terms were assigned and enriched using GO (http://www.geneontology.org/, accessed on 2 February 2025), and the pathway enrichment analysis was conducted via the KEGG database. For both GO and KEGG analyses (http://www.genome.jp/kegg/, accessed on 2 February 2025), a Q-value ≤ 0.05 was set as the threshold for statistically significant enrichment. All DEGs and detailed enrichment results are provided in Supplementary Tables S2 and S3.

### 2.5. RNA Extraction and Real-Time RT-PCR

The total RNA was extracted using HiPure RNA Kit (Magen, Guangzhou, China). RNA (2 μg) was reverse transcribed into cDNA by a Reverse transcription kit (TAKARA, Kusatsu, Shiga, Japan). Genes of interest and the internal control (*β-Actin*) were amplified using SYBR Green I Master Mix (Roche, Indianapolis, IN, USA) in LightCycler 480 (Roche, USA) platform. Each gene was amplified in triplicate. The relative amplification of genes was calculated using 2^−ΔΔCT^ method.

### 2.6. Statistical Analysis

All data have at least three biological replicates and are presented as the mean ± standard deviation (SD). Statistical differences between wheat treated with and without γ-PGA were analyzed using *t*-test and Duncan’s tests of one-way ANOVAs in SPSS (version 22.0.0.0). Significant differences are indicated by asterisks: * *p* < 0.05; ** *p* < 0.01.

## 3. Results

### 3.1. Exogenous γ-PGA Application Can Significantly Enhance the Cold Resistance and Frost Tolerance of Wheat

**Figure 1:**
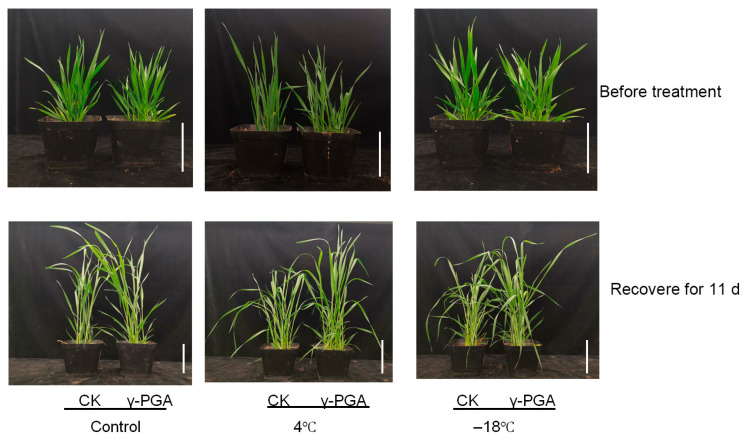
Phenotypic effects of exogenous γ-PGA application on chilling (4 °C) and freezing (−18 °C) resistance in wheat. (bar = 10 cm).

**Figure 2:**
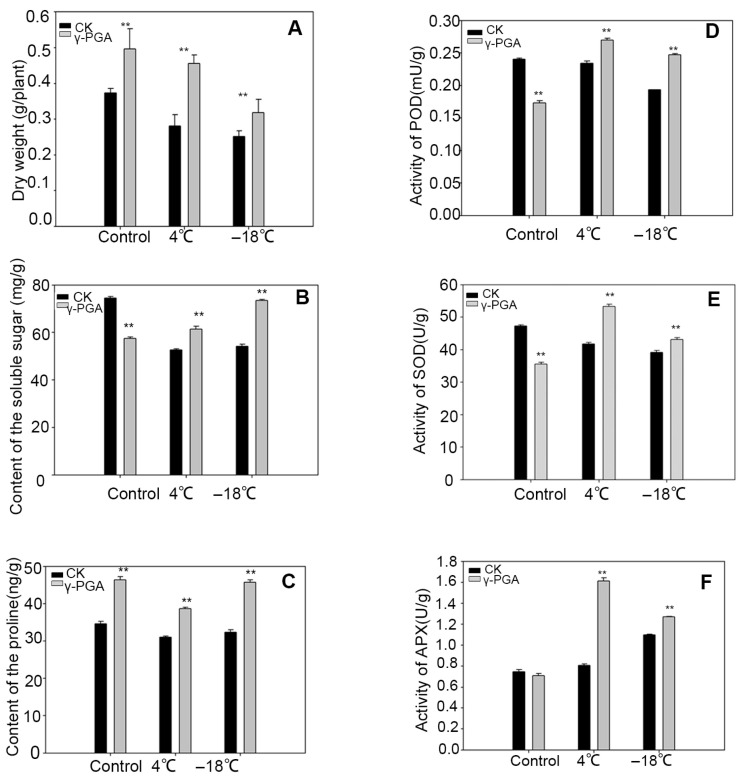
Effects of cold stress on dry weight and key physiological parameters in wheat leaves. (**A**) Dry weight of wheat leaves under control, chilling (4 °C), and freezing (−18 °C) stress conditions. (**B**,**C**) Contents of soluble sugar (**B**) and proline (**C**) in wheat leaves under control, chilling, and freezing stress conditions. (**D**–**F**) Activities of peroxidase (POD, (**D**)), superoxide dismutase (SOD, (**E**)), and ascorbate peroxidase (APX, (**F**)) in wheat leaves with or without γ-PGA treatment under normal, chilling (4 °C, 24 h), and freezing (−18 °C, 3 h) conditions. Values are means ± sd (n ≥ 3 repeats). Significant differences are indicated by asterisks (** *p* ≤ 0.01).

To investigate the effects of exogenous γ-PGA on wheat cold resistance, we conducted experiments on wheat seedlings’ frost tolerance and cold resistance during the seedling stage. The results of the wheat seedling freezing tolerance experiment showed that after 12 h of exposure to −18 °C, the survival rate of seedlings in the untreated control group was only 26.80%. In contrast, the survival rate in the group treated with exogenous γ-PGA was significantly higher, reaching approximately 61.3% (2.29 times that of the control). This demonstrates a pronounced enhancement of freezing tolerance by γ-PGA treatment (Figure S1). This indicated that under low-temperature stress conditions, the wheat seeds treated with γ-PGA exhibited a significant increase in germination rate. This suggests that exogenous application of γ-PGA can significantly enhance the cold resistance of wheat plants. The cold resistance experiment during the seedling stage involved low-temperature treatment at 4 °C and freezing treatment at −18 °C, with wheat grown under normal conditions serving as the control group. Following recovery under normal growth conditions after cold stress (4 °C and −18 °C), the control wheat seedlings exhibited significant growth inhibition, with more severe damage and pronounced leaf wilting observed after freezing stress. In contrast, the inhibition was less severe in the γ-PGA-treated seedlings. A significant growth advantage emerged in the γ-PGA-treated plants, which were noticeably taller than the controls after the 11-day recovery period (Figure 1). The results demonstrated that γ-PGA treatment alleviated the inhibitory effects of cold damage on wheat growth, regardless of whether the plants underwent low-temperature or freezing treatment. This was primarily evidenced by the significantly higher leaf dry weight in wheat plants treated with exogenous γ-PGA compared to the untreated group (Figure 2A). Under normal growth conditions, the dry weight of wheat treated with γ-PGA was 32.91% higher than that of the control. Under cold stress conditions, the wheat growth rate was markedly inhibited; however, γ-PGA treatment could significantly mitigate the impact of cold stress on wheat plants. Specifically, under low-temperature treatment at 4 °C and freezing treatment at −18 °C, the dry weight of wheat treated with γ-PGA was 62.44% and 26.56% higher than that of the control, respectively (Figure 2A). Additionally, we measured physiological indicators such as proline and soluble sugar content, as well as the activities of peroxidase (POD), superoxide dismutase (SOD) and ascorbate peroxidase (APX) wheat leaves (Figure 2B–F). Under low-temperature stress at 4 °C, the contents of proline and soluble sugar in wheat leaves treated with γ-PGA were significantly increased by 24.64% and 16.72%, respectively, compared with the control group. Under freezing stress at −18 °C, γ-PGA treatment also significantly promoted the accumulation of proline and soluble sugar, with increases of 42.26% and 35.58%, respectively (Figure 2B,C). Furthermore, γ-PGA treatment effectively activated the antioxidant enzyme system in wheat leaves. At 4 °C stress, the activities of POD, SOD and APX were increased by 15.12%, 27.73% and 99.37%, respectively, compared with the control. Under freezing stress at −18 °C, the activities of these three enzymes were also elevated by 27.82%, 10.33%, and 15.91%, respectively, relative to the control (Figure 2D–F). The results showed that γ-PGA treatment could significantly increase proline and soluble sugar content, as well as the activities of these enzymes in wheat leaves, thereby mitigating the damage caused by cold stress to wheat seedlings. These findings indicate that γ-PGA can effectively enhance wheat cold resistance and frost tolerance.

### 3.2. Differentially Expressed Genes (DEGs) Between γ-PGA-Treated and Control Wheat Under Chilling and Freezing Stress

**Figure 3:**
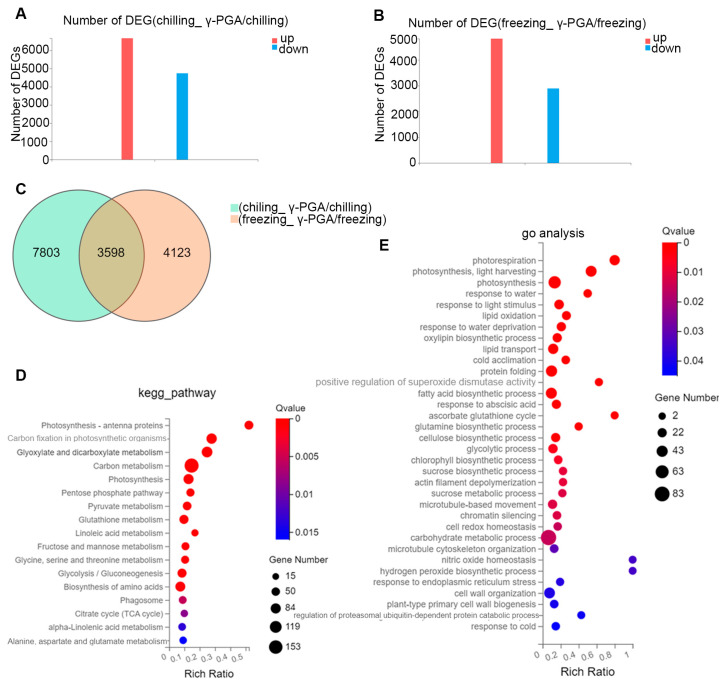
The differentially expressed genes (DEGs) and KEGG enrichment analysis of the DEGs—the overlap of DEGs between (chilling_γ-PGA/chilling) and (freezing_γ-PGA/freezing). (**A**) The number of DEGs (chilling_γ-PGA/chilling) identified by RNA sequence analysis. (**B**) The number of DEGs (freezing_γ-PGA/freezing) identified by RNA sequence analysis. (**C**) The Venn diagram of differentially expressed genes between DEGs (chilling_γ-PGA/chilling) and DEGs (freezing_γ-PGA/freezing). (**D**) The KEGG enrichment analysis of the overlap of DEGs between (chilling_γ-PGA/chilling) and (freezing_γ-PGA/freezing). (**E**) The Go enrichment analysis the overlap of DEGs between (chilling_γ-PGA/chilling) and (freezing_γ-PGA/freezing).

The leaves of the γ-PGA-treated and control wheat under cold stress conditions (chilling (4 °C) and freezing (−18 °C)) were used for RNA sequencing to identify the DEGs and pathways upregulated in response to cold stress. The total raw reads, clean reads, clean reads ratio, genome mapping ratio, gene mapping ratio, and unique mapping ratio are listed in Table S1. A total of 11,401 differentially expressed genes (DEGs) were identified in the comparison of (chilling_γ-PGA/chilling), among which 6664 genes were significantly upregulated and 4737 were significantly downregulated. In the comparison of (freezing_γ-PGA/freezing), a total of 7721 differentially expressed genes (DEGs) were identified, among which 4828 genes were significantly upregulated and 2893 were significantly downregulated (Figure 3A,B). The Venn diagram illustrates the overlap of differentially expressed genes between (chilling_γ-PGA/chilling) and (freezing_γ-PGA/freezing). Among these, 3598 genes are commonly differentially expressed (Figure 3C).

### 3.3. KEGG and GO Pathway Analysis of the Overlapping DEGs Under Chilling and Freezing Stress

In total, 3598 genes were commonly differentially expressed under both chilling and freezing stress, and these DEGs were subjected to KEGG pathway and Gene Ontology (GO) function enrichment analysis. Based on the KEGG pathway analysis, all of the DEGs were mainly significantly enriched in 13 pathways (Q value ≤ 0.01). Pathways significantly enriched were predominantly related to photosynthesis, carbon fixation, glyoxylate and dicarboxylate metabolism, pentose phosphate pathway glutathione metabolism, linoleic acid metabolism, fructose and mannose metabolism, and amino acid biosynthesis (Figure 3D).

GO functional analysis further elucidated the biological processes modulated by γ-PGA. The overlapping DEGs were significantly enriched in terms directly associated with cold acclimation, response to water deprivation/abscisic acid, redox homeostasis (e.g., superoxide dismutase activity), and the biosynthesis of protective compounds such as fatty acids, oxylipins, and cell wall components (Q value ≤ 0.05) (Figure 3E). This integrated profile suggests that γ-PGA priming enhances wheat’s cold tolerance by simultaneously bolstering energy metabolism, activating antioxidant systems, and reinforcing cellular structures (Figure 3E).

### 3.4. GO Analysis of the DEGs Identified in Response to Chilling and Freezing Stresses

**Figure 4:**
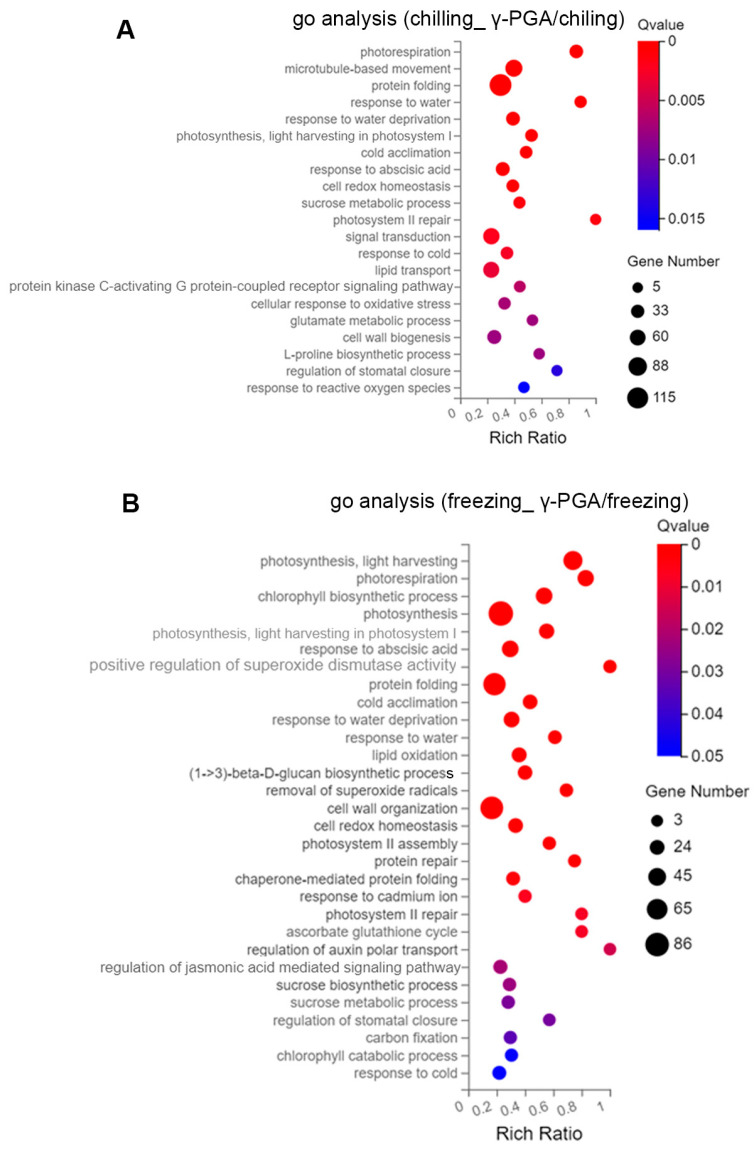
GO enrichment analysis of the DEGs—the differentially expressed genes identified under chilling and freezing stresses in the two respective groups. (**A**) Go analysis of the DEGs identified under chilling stress; (**B**) Go analysis of the DEGs identified under freezing stress.

To dissect the distinct effects of γ-PGA under different stress intensities, we performed separate GO analyses for chilling- and freezing-specific DEGs (Figure 4). Under chilling stress, γ-PGA-triggered genes were notably enriched in processes involving microtubule-based movement, protein folding, response to water deprivation, photorespiration, cold acclimation, cellulose biosynthetic process, response to abscisic acid (33 DEGs); oxylipin biosynthetic process, cell redox homeostasis, cell wall organization, fatty acid biosynthetic process, L-arabinose metabolic process, sucrose metabolic process, ascorbate glutathione cycle, response to cold, lipid transport, cellular response to oxidative stress, glutamate metabolic process and L-proline biosynthesis (Q value ≤ 0.01) (Figure 4A), pointing to early adjustments in cellular architecture and metabolism. In contrast, under the more severe freezing stress, the response strongly emphasized photosystem protection, chaperone-mediated protein folding, positive regulation of superoxide dismutase activity, cold acclimation, removal of superoxide radicals, cell wall organization, cell redox homeostasis, cellulose biosynthetic process, microtubule-based movement, response to cadmium ion, ascorbate glutathione cycle and the regulation of stress-signaling pathways like jasmonic acid signaling (Q value ≤ 0.01) (Figure 4B). This divergence highlights that γ-PGA’s protective mechanism is dynamically tailored to the severity of the low-temperature stress.

### 3.5. γ-PGA Treatment Significantly Altered the Expression of Related Genes in Primary Carbon and Lipid Metabolism Pathways Under Chilling and Freezing Stress

**Figure 5:**
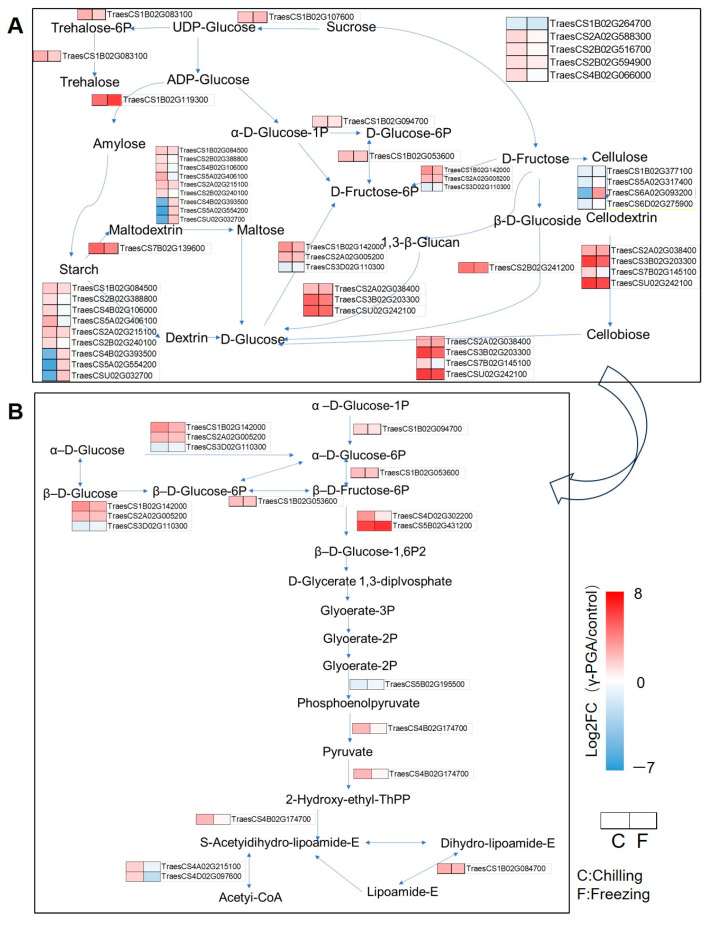
Expression analysis of the DEGs—the overlap of DEGs between (chilling_γ-PGA/chilling) and (freezing_γ-PGA/freezing) in starch and sucrose metabolism and glycolysis/gluconeogenesis pathway. (**A**) Starch and sucrose metabolism pathway. (**B**) Glycolysis/gluconeogenesis pathway. The heatmap in red and blue indicates gene expression abundance.

The transcriptomic analysis revealed that the γ-PGA treatment induced significant changes in the expression of multiple genes in wheat carbohydrate metabolism pathways under both chilling and freezing stress. These pathways included starch and sucrose metabolism, as well as glycolysis/gluconeogenesis. Within the starch and sucrose metabolism pathway, the γ-PGA treatment strongly induced the expression of several key enzyme genes (Figure 5A); for example, the expression of the starch synthase gene *TraesCS1B02G119300* was significantly induced under both chilling and freezing stress, with log_2_FC values of 5.64 and 7.33, respectively. The expression of the glucoamylase genes (EC 3.2.1.39) *TraesCS1A02G422900* and *TraesCS2B02G241200* was also significantly upregulated by γ-PGA. Furthermore, the β-amylase gene *TraesCS2D02G221000* (EC 3.2.1.2) and the isoamylase gene *TraesCS7D02G546800* (EC 3.2.1.4) showed sustained upregulation following γ-PGA treatment under both stress conditions. The expression of some genes exhibited opposite trends in response to the γ-PGA treatment under different stress types; for example, sucrose synthase-related genes (EC 2.4.1.13), including *TraesCS2A02G406700*, *TraesCS2B02G424300*, and *TraesCS2D02G403600*, were slightly upregulated by γ-PGA under chilling stress but were downregulated under freezing stress. The gene *TraesCS5B02G318000* (EC 3.2.1.4) showed a slight downregulation (log_2_FC = −0.72) after PGA treatment under chilling stress, while it was induced and upregulated (log_2_FC = 1.92) under freezing stress. In the glycolysis and gluconeogenesis pathways, the γ-PGA treatment also induced the upregulation of several genes (Figure 5B). The hexokinase gene *TraesCS1B02G142000* (EC 2.7.1.1) showed log_2_FC values of 3.48 and 2.38 under chilling and freezing stress, respectively. The expression of the 6-phosphofructokinase gene *TraesCS5B02G431200* (EC 2.7.1.11) was strongly induced by γ-PGA, with log_2_FC values reaching 5.93 and 6.40. The expression of the glucose-6-phosphate isomerase gene *TraesCS1B02G053600* (EC 5.3.1.9) and the phosphoglucomutase gene *TraesCS1B02G094700* (EC 5.4.2.2) was consistently upregulated following the γ-PGA treatment. Additionally, the expression of several alcohol dehydrogenase genes (EC 1.1.1.2), including *TraesCS1A02G298200*, *TraesCS1B02G307700*, and *TraesCS1D02G293000*, was upregulated by γ-PGA under both stress conditions. The expression of the pyruvate dehydrogenase gene *TraesCS7B02G103800* (EC 1.2.4.1) and the dihydrolipoamide dehydrogenase gene *TraesCS1B02G084700* (EC 1.8.1.4) was also induced by γ-PGA.

**Figure 6:**
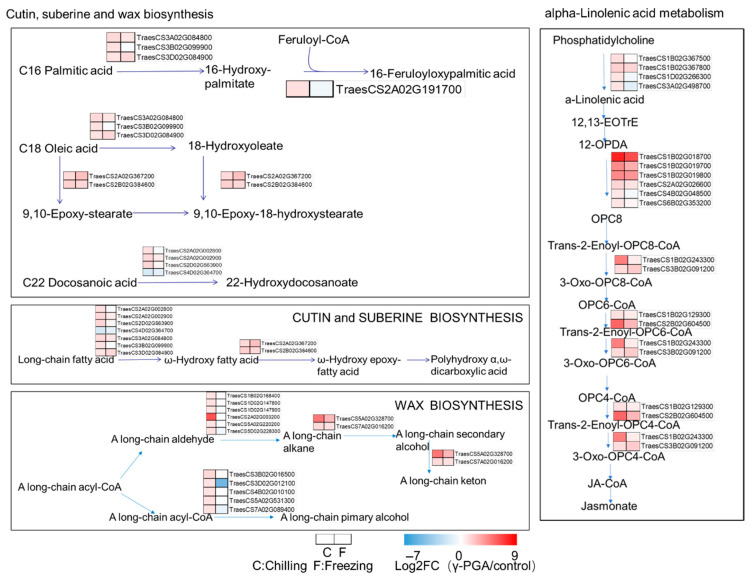
Expression analysis of the DEGs—the overlap of DEGs between (chilling_γ-PGA/chilling) and (freezing_γ-PGA/freezing) in Cutin, suberine and wax biosynthesis pathway and alpha-Linolenic acid metabolism. The heatmap in red and blue indicates gene expression abundance.

The transcriptomic analysis revealed that 30 genes involved in cutin, suberin, and wax biosynthesis and 20 genes related to α-linolenic acid metabolism were differentially expressed after γ-PGA treatment under both chilling and freezing stress (Figure 6). In the former pathway, the γ-PGA treatment upregulated most genes under both chilling and freezing stress. Among them, the expression of *TraesCS2A02G003200* was most strongly induced under chilling stress, with a log_2_FC of 6.11. Also, *TraesCS5A02G328700* was strongly upregulated by γ-PGA under both types of stress (log_2_FC = 4.88 and 2.60, respectively). *TraesCS2B02G384600* was also consistently upregulated in response to γ-PGA (chilling: log_2_FC = 1.76; freezing: log_2_FC = 1.47). In contrast, the γ-PGA treatment led to the downregulation of some genes, such as *TraesCS1D02G147900*, *TraesCS2A02G002800,* and *TraesCS2A02G191700*, under freezing stress. In the α-linolenic acid metabolic pathway, the γ-PGA treatment strongly induced the expression of multiple genes under stress conditions. For example, *TraesCS1B02G018700* exhibited the highest upregulation after γ-PGA application, with log_2_FC values of 8.52 and 7.06 under chilling and freezing stresses, respectively. Its paralogs *TraesCS1B02G019700* and *TraesCS1B02G019800* were also markedly upregulated by γ-PGA. The expression of *TraesCS2B02G604500* was significantly induced by γ-PGA under chilling stress (log_2_FC = 6.04). Additionally, *TraesCS1B02G243300* was strongly upregulated by γ-PGA under chilling (log_2_FC = 5.09) but showed a reduced induction under freezing stress (log_2_FC = 0.85). Notably, the γ-PGA treatment modulated the expression of genes involved in multiple pathways; for instance, *TraesCS3A02G084800*, *TraesCS3B02G099900* and *TraesCS3D02G084900*, which participate in both fatty acid degradation and cutin/wax biosynthesis, were differentially expressed. Similarly, *TraesCS1B02G129300* and *TraesCS2B02G604500*, which are involved in α-linolenic acid metabolism, fatty acid degradation, and peroxisome-related processes, were also regulated by γ-PGA.

### 3.6. γ-PGA Strongly Enhances Genes Related to the Phenylpropanoid/Flavonoid Biosynthesis Pathway Under Both Chilling and Freezing Stress

**Figure 7:**
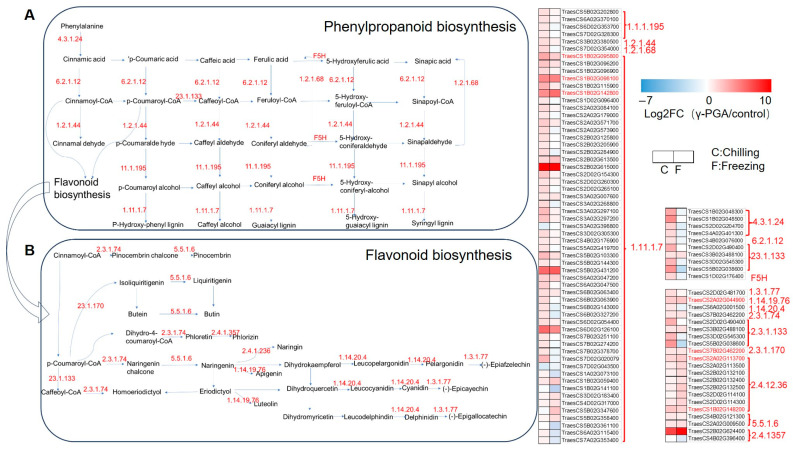
Expression analysis of the DEGs—the overlap of DEGs between (chilling_γ-PGA/chilling) and (freezing_γ-PGA/freezing) in phenylpropanoid biosynthesis and flavonoid biosynthesis pathway. (**A**) Phenylpropanoid biosynthesis pathway. (**B**) Flavonoid biosynthesis pathway. The heatmap in red and blue indicates gene expression abundance.

The transcriptome analysis revealed that the γ-PGA treatment induced strong and persistent activation of the phenylpropanoid/flavonoid biosynthesis pathway in wheat under both chilling and freezing stresses, leading to coordinated reprogramming of gene expression from the upstream general phenylpropanoid pathway to the downstream flavonoid-specific branches (Figure 7).

In the core phenylpropanoid pathway, the γ-PGA treatment consistently upregulated the expression of multiple key enzyme-encoding genes (Figure 7A). Upstream genes, including those encoding phenylalanine ammonia-lyase (PAL, EC 4.3.1.24), cinnamate 4-hydroxylase (C4H), and 4-coumarate-CoA ligase (4CL), were synchronously induced by γ-PGA, ensuring directed flux of carbon into phenylpropanoid metabolism. A notable finding was the extremely significant upregulation of multiple peroxidase-encoding genes (EC 1.11.1.7) by γ-PGA; for instance, the expression of *TraesCS2B02G615000* was increased by over 500- and 800-fold under γ-PGA treatment during chilling and freezing stress, respectively (log_2_FC: 9.57 and 9.73). As a key catalyst for lignin polymerization, its upregulation suggests an enhancement of cell wall lignification, potentially providing direct physical reinforcement in wheat. Under chilling stress, γ-PGA upregulated 77% of the core phenylpropanoid enzyme genes, with 14 peroxidase members showing log_2_FC values between 2.3 and 4.7. Under freezing stress, although some genes were downregulated, 42% of the genes maintained an upregulation of log_2_FC ≥ 0.5 in response to γ-PGA. The key nodal gene, *TraesCS5B02G431200*, situated at the intersection of sugar and phenylpropanoid metabolism, was strongly induced by γ-PGA under both stresses (log_2_FC: 5.93 and 6.40), effectively linking primary and secondary metabolism.

Genes related to the downstream flavonoid and stilbene biosynthesis branches were also significantly upregulated by γ-PGA treatment (Figure 7B). Coordinated induction was observed for genes encoding enzymes from upstream chalcone synthase (CHS, EC 2.3.1.170/2.3.1.74) to downstream enzymes such as chalcone isomerase (CHI, EC 5.5.1.6), anthocyanidin synthase (ANS, EC 1.14.19.76), and various flavonoid glycosyltransferases (UFGT, EC 2.4.1.236). For example, γ-PGA treatment increased the expression of the flavonoid glycosyltransferase gene *TraesCS2B02G132600* (log_2_FC: 1.47 and 4.11) and the stilbene synthase gene *TraesCS2D02G490400* (log_2_FC: 3.19 and 0). Furthermore, γ-PGA treatment induced stronger upregulation of most genes, including *TraesCS2B02G132600* (*UFGT*), *TraesCS2A02G113700* (*UFGT*), *TraesCS2A02G044900* (*ANS*), and *TraesCS1B02G148200* (*CHI*), under freezing stress compared to chilling stress.

### 3.7. γ-PGA Could Enhance the Expression of Genes in Antioxidant Systems Including Glutathione Metabolism and Peroxidase Synthesis Under Both Chilling and Freezing Stress

**Figure 8:**
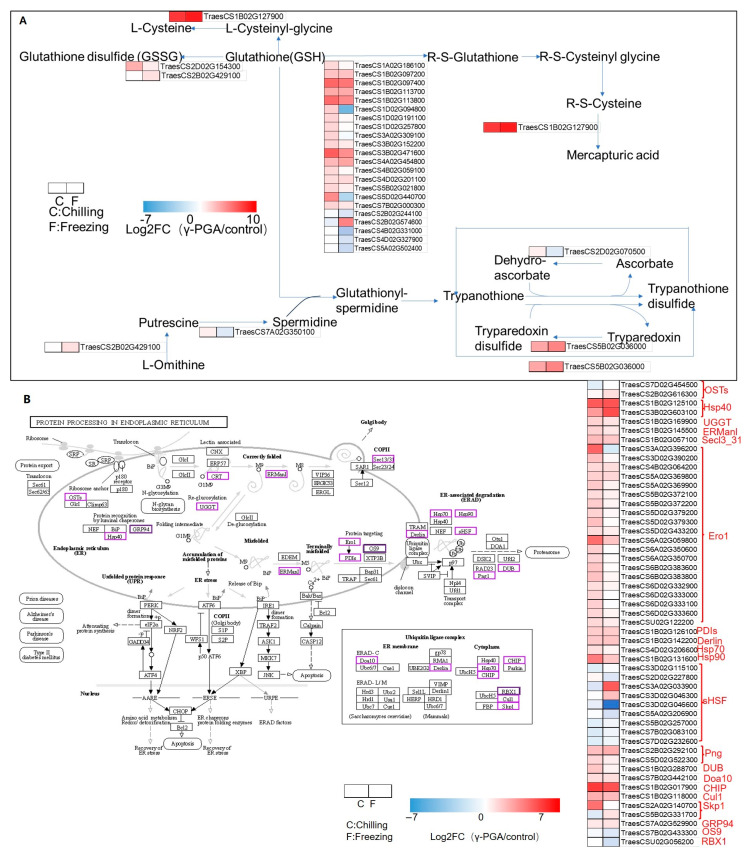
Expression analysis of the DEGs—the overlap of DEGs between (chilling_γ-PGA/chilling) and (freezing_γ-PGA/freezing) in protein processing in endoplasmic reticulum and glutathione metabolism pathway. (**A**) Glutathione metabolism pathway. The heatmap in red and blue indicates gene expression abundance. (**B**) Protein processing in endoplasmic reticulum.

The transcriptome analysis revealed that the γ-PGA treatment significantly induced the expression of multiple genes within the glutathione metabolism pathway in wheat under both chilling and freezing stress (Figure 8A). Specifically, the gene *TraesCS1B02G127900*, encoding a peptidase, exhibited the most pronounced upregulation in response to γ-PGA, with log_2_FC values of 5.42 and 6.11 under chilling and freezing conditions, respectively. Several glutathione S-transferase (GST) genes were also markedly induced by γ-PGA under both stress regimes, including *TraesCS1B02G113800* (log_2_FC: 4.10 and 3.26) and *TraesCS3B02G471600* (log_2_FC: 4.47 and 3.12).

Furthermore, the γ-PGA treatment dramatically increased the expression of multiple peroxidase-encoding genes, with the most prominent induction observed for *TraesCS2B02G615000*, with log_2_FC values reaching 9.57 and 9.73 following γ-PGA application under chilling and freezing stress, respectively. The expression of genes involved in the synthesis of antioxidant secondary metabolites was also enhanced by γ-PGA, including the flavonoid glycosyltransferase gene *TraesCS2B02G132600* (log_2_FC: 1.47 and 4.11). Additionally, γ-PGA significantly upregulated the expression of *TraesCS2B02G604500* (log_2_FC: 6.04 and 2.89), an ACOX gene involved in fatty acid β-oxidation, along with other genes associated with peroxisome function. These results indicate that γ-PGA may synergistically enhance the plant’s antioxidant capacity by inducing the expression of genes across multiple interconnected defense systems in response to chilling and freezing stress.

### 3.8. γ-PGA Significantly Regulates the Expression of Genes in the Protein Processing Pathway in the Endoplasmic Reticulum of Wheat Under Chilling and Freezing Stress

The γ-PGA treatment significantly regulated the expression of genes related to the protein processing pathway in the endoplasmic reticulum (ER) of wheat (Figure 8B). The results showed that the vast majority of protein processing pathway related genes (approximately 85%) were significantly upregulated. Among them, genes encoding the molecular chaperone Hsp40 (e.g., *TraesCS1B02G125100* and *TraesCS3B02G603100*) exhibited extremely significant upregulation, with log_2_FC values of 5.21 and 3.20 under chilling stress, and 5.39 and 5.59 under freezing stress, respectively. Simultaneously, the gene encoding the key ER oxidoreductase Ero1 (e.g., *TraesCS3A02G396200*) was strongly induced under chilling stress (log_2_FC = 4.92). Furthermore, the expression of other genes related to ER protein quality control, such as those encoding UGGT, ERManI, Secl3_31, PDIs, and Derlin, was generally upregulated. Notably, the expression of the OST gene (*TraesCS7D02G454500*) was suppressed.

Finally, to confirm the RNA-seq results, nine genes with significant transcript abundances were selected for RT-PCR validation (Figure S2). These genes function in several key pathways: phenylpropanoid-flavonoid metabolism (*TraesCS2B02G615000*, *TraesCS2B02G624400*), glutathione metabolism (*TraesCS1B02G127900*, *TraesCS5B02G021800*), lipid metabolism (*TraesCS1B02G018700*), MAPK signaling pathway (*TraesCS1B02G100300*), starch and sucrose metabolism (*TraesCS7B02G139600*, *TraesCS1B02G119300*), and pentose and glucuronate interconversions (*TraesCS5B02G431200*).

## 4. Discussion

Low temperature is a primary abiotic stress that severely restricts wheat growth and yield, especially during vulnerable developmental stages threatened by late spring cold spells [7]. Therefore, enhancing cold tolerance is of paramount agronomic importance. This study demonstrates that exogenous application of γ-PGA effectively mitigates the growth inhibition caused by both chilling (0–15 °C) and freezing (<0 °C) stress in wheat seedlings. While γ-PGA has shown promise in improving plant stress resistance, its specific mechanisms in enhancing low-temperature tolerance remain unclear. By integrating physiological assays with transcriptomic profiling, we propose that γ-PGA bolsters wheat cold resilience through a coordinated strategy centered on enhancing antioxidant capacity, reinforcing physical barriers, and sustaining energy metabolism, with the phenylpropanoid-flavonoid pathway playing a particularly pivotal role.

The low-temperature treatment employed in this study was primarily designed to simulate short-term, severe cold snaps that wheat may encounter in early spring or late autumn. While temperature fluctuations in the field are typically more gradual, sudden and sharp drops in temperature—which often cause irreversible damage to plants—are critical scenarios for assessing a cultivar’s cold hardiness. By setting controlled freezing intensity and duration, we aimed to systematically compare the physiological and molecular responses under different treatments, which helps elucidate the mechanism by which γ-PGA enhances freezing tolerance. We plan to conduct field experiments in the future to validate these laboratory findings. And future studies should validate these findings across a broader range of genotypes to ensure the reliability and reproducibility of the conclusions.

Physiological and biochemical assays revealed that γ-PGA significantly increased the contents of soluble sugars and proline, as well as peroxidase (POD) activity in wheat under both chilling and freezing stress. This indicates that γ-PGA enhances wheat chilling and freezing tolerance by promoting the accumulation of osmoprotectants and boosting the plant’s antioxidant capacity. Here, we combined physiological assays with transcriptome profiling to dissect how γ-PGA enhances wheat cold and freezing tolerance. The transcriptomic analysis revealed a complex molecular network by which γ-PGA modulates wheat responses to 4 °C chilling and –18 °C freezing. γ-PGA altered the expression of thousands of genes under both types of stress, with more differentially expressed genes (DEGs) under chilling (11,401) than under freezing stress (7721), suggesting that moderate cold initiates broader adaptive programs, whereas extreme cold may repress or damage certain pathways. Notably, 3598 DEGs were shared between the two stresses, likely comprising a core regulatory network underlying γ-PGA-mediated low-temperature resilience. Accordingly, it is postulated that γ-PGA may elevate the cold resistance of wheat via the mechanisms outlined below.

### 4.1. γ-PGA Boosts Antioxidant Defense and Osmoprotection (Core Mechanism)

Low-temperature stress can induce the production of large amounts of ROS in plants, leading to lipid peroxidation and oxidative damage to proteins and genetic materials, as well as cell death [28]. Excessive ROS can cause cytotoxicity by oxidizing biomacromolecules such as proteins, nucleic acids, and membrane lipids, disrupting normal cellular metabolic functions. To cope with this oxidative stress, plants activate a sophisticated antioxidant defense system that involves the coordinated action of antioxidant enzymes such as peroxidase (POD), catalase (CAT), and superoxide dismutase (SOD), as well as the accumulation of osmoregulatory substances like proline, collectively maintaining redox homeostasis [29]. Physiological assays confirmed that γ-PGA significantly increased SOD, POD, and APX activities, implying that γ-PGA activates ROS-scavenging systems to preserve membrane integrity. Furthermore, transcriptomic evidence showed that γ-PGA upregulated genes encoding these antioxidant enzymes; in particular, the “positive regulation of superoxide dismutase activity” was enriched under freezing stress, paralleling the observed increases in SOD and POD activities in Figure 2. γ-PGA also upregulated a series of genes in the phenylpropanoid–flavonoid metabolic pathway, among which the genes encoding peroxidases (POD, EC 1.11.1.7) all exhibited significant upregulation in Figure 7. Notably, the expression of the peroxidase gene *TraesCS2B02G615000* showed the most remarkable increase, with log_2_FC values reaching 9.57 under chilling stress and 9.73 under freezing stress, indicating that its expression level increased by several-hundred-fold, which is consistent with the observed increase in peroxidase (POD) activity in the wheat leaves. And RT-PCR validation confirmed that *TraesCS2B02G615000* was significantly induced by γ-PGA under both cold and freezing stress conditions. The activation of this pathway directly points to enhanced cell wall lignification, providing physical reinforcement for cells. Meanwhile, the downstream branch of this pathway (the flavonoid and stilbene biosynthesis pathways) was also coordinately induced, suggesting the accumulation of secondary antioxidant metabolites [30]. Additionally, the significant upregulation of GST genes and multiple peroxidase-encoding genes, coupled with the simultaneous activation of the phenylpropanoid–flavonoid pathway, collectively established both enzymatic and non-enzymatic antioxidant systems that effectively maintain cellular redox homeostasis. This finding is highly consistent with the enrichment results of pathways such as “cellular redox homeostasis” and “positive regulation of superoxide dismutase activity” in the GO analysis. The widespread upregulation of genes related to the protein processing pathway in the endoplasmic reticulum (ER), such as the molecular chaperone *Hsp40* and the oxidoreductase *Ero1*, suggests that γ-PGA helps alleviate ER stress and protein misfolding caused by low temperatures, thereby maintaining cellular proteostasis. This effect, in synergy with other antioxidant systems—such as the induced flavonoid and stilbene biosynthesis pathways—effectively clears excess reactive oxygen species (ROS) that have accumulated under cold stress, preventing oxidative damage to membrane lipids, proteins, and nucleic acids.

Additionally, the upregulation of genes related to proline and sucrose synthesis further supports the hypothesis that γ-PGA promotes the accumulation of osmoprotective substances to maintain cellular homeostasis. Physiological and biochemical assays also confirmed that γ-PGA treatment significantly increases the proline and soluble sugar content in wheat leaves under both chilling and freezing stresses, consistent with the transcriptomic analysis results in Figure 2. Soluble sugars not only are important osmoregulatory substances but also stabilize membrane structures and proteins through hydrogen bonding, preventing ice crystal damage [31].

### 4.2. γ-PGA May Sustain Energy Supply via Carbon-Metabolism Regulation

The KEGG enrichment analysis showed significant over-representation of photosynthesis-related pathways (e.g., photosystem antenna proteins and carbon fixation) and carbon metabolism (glycolysis and pentose phosphate pathway). The reprogramming of energy metabolism and substance synthesis provides a material and energy basis for the cold resistance process. The upregulation of key genes in central carbon metabolism pathways, such as glycolysis, the pentose phosphate pathway, and starch–sucrose metabolism, not only ensures a continuous energy supply but also provides precursors for the synthesis of proline and soluble sugars. This directly explains the physiological phenomenon of higher levels of osmotic adjustment substances (soluble sugars and proline) in the γ-PGA treatment group in Figure 2.

### 4.3. γ-PGA May Enhance the Cold Resistance of Wheat by Strengthening Physical Barriers and Cellular Structures

The cuticle, a protective layer covering the outer epidermal cell walls of above-ground plant organs, consists primarily of a cutin matrix embedded in and sealed with cuticular waxes. It plays a crucial role in plants’ adaptation to extreme temperature fluctuations [32]. The γ-PGA treatment significantly upregulated the expression of multiple genes involved in cutin, wax, and suberin biosynthesis. The deposition of these hydrophobic lipids on the plant epidermis can effectively reduce non-stomatal water loss and form a physical barrier under low-temperature conditions, preventing ice crystal intrusion and damage caused by extracellular freezing [32]. Simultaneously, the phenylpropanoid metabolic pathway was strongly and persistently activated. Notably, the significant upregulation of genes encoding peroxidases (e.g., *TraesCS2B02G615000*, with an expression increase in several-hundred-fold) and enzymes involved in lignin monomer synthesis (PAL, C4H, and 4CL) likely collectively drives cell wall reinforcement and lignification. This provides direct mechanical support for cells and enhances tissue resistance to cellular dehydration caused by freezing and the osmotic stress that occurs following thawing.

Studies have indicated that γ-PGA treatment strongly induces the α-linolenic acid metabolic pathway. This pathway is central to the biosynthesis of jasmonic acid (JA), which, as a crucial stress hormone, can further amplify defense signals [33,34] and globally regulate the expression of numerous stress-responsive genes, including the aforementioned wax synthesis and phenylpropanoid metabolism genes, forming a positive feedback regulatory loop. Furthermore, genes involved in fatty acid β-oxidation (e.g., *TraesCS2B02G604500*) were also upregulated, which may help mobilize lipids as an energy source during stress and participate in the formation of reactive oxygen species (ROS) signaling. The remodeling of lipid metabolism not only releases signaling molecules but may also enhance plant cold resistance by adjusting membrane lipid unsaturation to maintain membrane fluidity. When temperatures drop, cell membranes transition from a liquid phase to a gel phase, leading to dysfunction of membrane proteins and triggering a series of physiological and morphological changes. During this process, changes in the composition of membrane lipid fatty acids are particularly critical, as they directly influence membrane fluidity and integrity [35].

In conclusion, the transcriptomic and physiological evidence converges into a coherent model. γ-PGA does not merely trigger a generic stress response but initiates a prioritized and integrated adaptive strategy. The most pronounced effect is the potent induction of the phenylpropanoid-flavonoid pathway, which directly enhances antioxidant capacity (via PODs and flavonoids) and cell wall strength (via lignification). This core defense is synergistically supported by the reinforcement of waxes and the maintenance of cellular homeostasis through energy and osmolyte production (soluble sugars, proline). The validation of key genes from these pathways (e.g., *TraesCS2B02G615000*, *TraesCS7B02G139600*) by RT-PCR (Figure S2) solidifies the credibility of this molecular model. This multifaceted mechanism elucidates how γ-PGA enhances wheat’s tolerance to both chilling and freezing stress, providing a robust theoretical foundation for its application in sustainable agriculture to combat low-temperature damage.

## 5. Conclusions

γ-PGA significantly enhances wheat chilling and freezing tolerance through an integrated mechanism involving osmolyte accumulation, antioxidant defense, and remodeling of cold-responsive gene networks—including phenylpropanoid–flavonoid, glutathione, and lipid metabolism—thus providing a mechanistic basis for its application in alleviating low-temperature stress in wheat production.

## Data Availability

Data supporting the findings of this work are available within the manuscript and its Supplementary Materials. The RNA-seq datasets are available from the National Center for Biotechnology Information: https://www.ncbi.nlm.nih.gov/sra/PRJNA1238059 (accessed on 19 March 2025). We used the following pathway databases: Gene Ontology (http://www.geneontology.org/) and KEGG (https://www.genome.jp/kegg, accessed on 2 February 2025).

## Supplementary Materials

The following supporting information can be downloaded at: https://www.mdpi.com/article/10.3390/biology15030293/s1. Figure S1. Effect of γ-PGA treatment on wheat seedling survival rate under freezing stress. Figure S2. Validation of the DEGs identified in RNA-seq by real-time RT-PCR. Table S1. The overall of RNAseq in this paper. Table S2. The DEGs between chilling_γ-PGA and chilling. Table S3. The DEGs between freezing_PGA and freezing.
