# Supplementary material for: Transcriptome Sequencing Analysis Reveals the Mechanisms of Poly-γ-Glutamic Acid Enhanced the Chilling and Freezing Tolerance in Wheat
Source: Biology (Basel). 2026 Feb 6;15(3):293. doi: 10.3390/biology15030293 (PMC12897291; doi:10.3390/biology15030293)
Supplement: Supplementary file 1 [file biology-15-00293-s001.zip › Fig.S1.pdf]

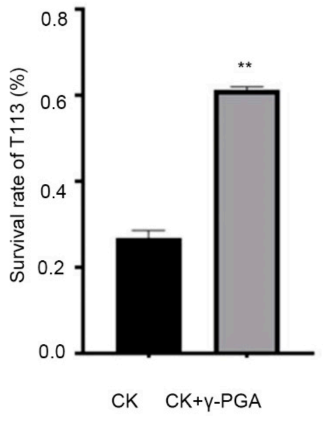


**Fig.S1 Effect of γ-PGA treatment on wheat seedling survival rate under freezing stress**

Values are means ± sd (n ⩾3 repeats). Significant differences are indicated by asterisks (**, P ⩽ 0.01).
