# Supplementary material for: Transcriptome Sequencing Analysis Reveals the Mechanisms of Poly-γ-Glutamic Acid Enhanced the Chilling and Freezing Tolerance in Wheat
Source: Biology (Basel). 2026 Feb 6;15(3):293. doi: 10.3390/biology15030293 (PMC12897291; doi:10.3390/biology15030293)
Supplement: Supplementary file 1 [file biology-15-00293-s001.zip › Fig.S2.pdf]

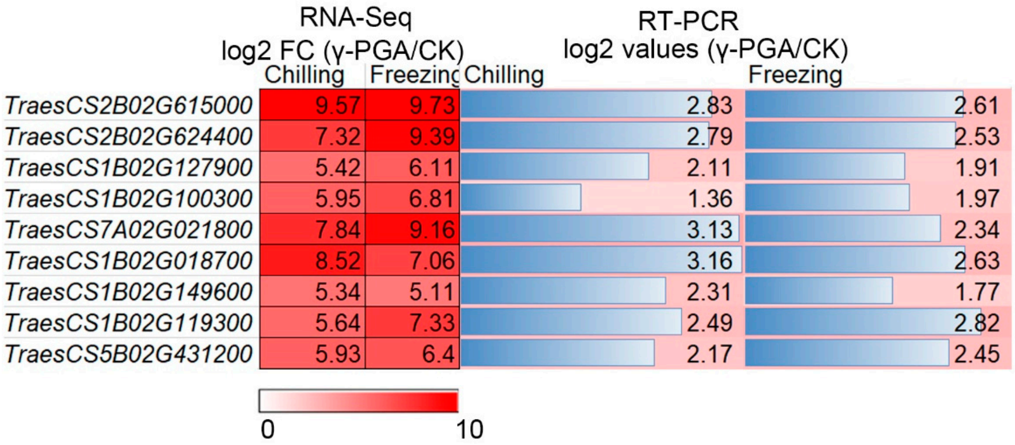

Fig. S2 Validation of the DEGs identified in RNA-seq by real-time RT-PCR

The left heatmap shows the log2 fold change of the DEGs identified in RNA-seq. The right bar graph shows the real-time RT-PCR results. Log2 values (γ-PGA /CK) were used to generate the plots. Real-time RT-PCR analyzed expression levels of genes, and fold changes in transcripts were calculated by the $2^{-\Delta\Delta Ct}$ method with Actin as the internal control.
