# Supplementary material for: Transcriptome Sequencing Analysis Reveals the Mechanisms of Poly-γ-Glutamic Acid Enhanced the Chilling and Freezing Tolerance in Wheat
Source: Biology (Basel). 2026 Feb 6;15(3):293. doi: 10.3390/biology15030293 (PMC12897291; doi:10.3390/biology15030293)
Supplement: Supplementary file 1 [file biology-15-00293-s001.zip › Table S1.pdf]

Table S1. The overall of RNAseq in this paper

Sample	Total Raw Reads (M)	Total Clean Reads (M)	Clean Reads Ratio(%)	Genome mapping(%)	Gene mapping(%)	Uniquely Mapping(%)
Chilling_1	82.14	75.01	91.33	97.23	78.07	92.45
Chilling_2	82.14	74.89	91.18	97.29	78.22	92.53
Chilling_3	82.14	75.10	91.43	97.28	78.13	92.68
Chilling_PGA _1	70.53	64.59	91.58	97.38	78.20	92.69
Chilling_PGA _2	82.14	75.00	91.31	97.39	78.13	92.63
Chilling_PGA _3	82.14	74.59	90.81	97.33	78.18	92.79
Freezing_1	82.14	75.26	91.63	97.31	77.86	92.55
Freezing_2	82.14	75.17	91.52	97.18	77.48	92.49
Freezing_2	80.92	73.36	90.65	97.29	77.74	92.75
Freezing_ PGA _1	82.14	75.32	91.69	97.37	77.25	92.67
Freezing_ PGA _2	82.14	75.24	91.60	97.33	77.34	92.85
Freezing_ PGA _3	80.39	74.64	92.85	97.43	77.31	92.74
